# Gut Microbiota Alterations and Primary Glomerulonephritis in Children: A Review

**DOI:** 10.3390/ijms24010574

**Published:** 2022-12-29

**Authors:** Anna Kawalec, Katarzyna Kiliś-Pstrusińska

**Affiliations:** Clinical Department of Paediatric Nephrology, Wroclaw Medical University, 50-556 Wroclaw, Poland

**Keywords:** glomerulnephritis, nephrotic syndrome, nephropathy IgA, microbiota, dysbiosis, children

## Abstract

The article summarizes the current evidence on the impact of microbiota alterations on immune-mediated primary glomerulonephritis in children. In particular, the focus is on the link between dysbiosis and the onset or recurrence of idiopathic nephrotic syndrome, immunoglobulin A nephropathy, and membranous nephropathy. The aim is to describe possible pathomechanisms, differences in gut microbiota composition between pediatric patients and healthy controls, and possible usage of microbiota manipulations in supportive therapy. On this basis, we attempt to indicate directions for further research in that field.

## 1. Introduction

The possible role of gut microbiota in the development of different diseases has been studied intensively over the last decades [1,2,3]. The interplay between residual intestinal bacteria and extra-intestinal organs is suggested to be one of the elements that may play a role in the pathogenesis of various immune-mediated diseases, including primary glomerulopathies [3,4].

Changes in the composition and function of gut microbiota may influence immune-mediated disease onset or recurrence by several mechanisms, including alteration of intestinal barrier function, increased oxidative stress, dysregulation of local and systemic inflammation [2], and impact on the differentiation and function of T regulatory cells (Treg) [5].

Although there are several studies investigating the influence of dysbiosis on glomerular diseases in adults [6,7,8], their number in the pediatric population is rather limited.

The article summarizes the current evidence on the impact of microbiota alterations on immune-mediated primary glomerulonephritis in children. In particular, the focus is on the link between dysbiosis and the onset or recurrence of idiopathic nephrotic syndrome, immunoglobulin A nephropathy, and membranous nephropathy. The aim is to describe possible pathomechanisms, differences in gut microbiota composition between pediatric patients and healthy controls, and possible usage of microbiota manipulations in supportive therapy, with an attempt to indicate directions for further research in that field.

## 2. Dysbiosis and Immunity

The human gut microbiota formation begins immediately after birth and is shaped during the first few years of life [3,9]. Some research suggests that it starts even earlier, and the transition of maternal bacteria to the fetus may take place during pregnancy [10]. It is presumed that gut microbiota stabilizes and becomes similar to that of adults at the age of 3 years [11,12]; however, recent findings indicate that its development in some children may take longer [9].

Three phases of gut microbiota development have been proposed: the developmental phase with a gradual shift in phyla detected and alpha-diversity, during which Bifidobacterium spp. dominates, the transitional phase in which alpha-diversity continues to change and only *Bacteroidetes* and *Proteobacteria* continue to develop, and the stable phase [13]. The most dominant phyla in the stable phase are *Bacteroidetes* and *Firmicutes* followed by *Proteobacteria*, *Fusobacteria*, *Tenericutes*, *Actinobacteria*, and *Verrucomicrobia*, of which *Firmicutes* and *Bacteroidetes* represent 90% of the bacteria in the gut [1,14]. It is noteworthy that the proportion of each phylum and structure of intestinal microbiota differs according to geographical region due to diverse dietary and other lifestyle factors [14].

The crosstalk between intestinal bacteria, the immune system, and kidneys is complex and dynamic [15,16]. Diverse bacteria differently impact and regulate immunological system responses [15]. Bacterial DNA, RNA, proteins, and cell wall components are ligands for host immune cells’ receptors (Toll-like receptors, nucleotide oligomerization receptors, C-type lectin receptors, and RIG-1-like receptors) [17]. Additionally, metabolites produced by commensal bacteria affect the host immune system locally and systemically [3].

Among microbiota-derived metabolites are those produced by gut bacteria from dietary compounds (short-chain fatty acids (SCFAs), microbial tryptophan catabolites, trimethylamine-N-oxide), produced by the host and modified by gut microbiota (secondary bile acids), or produced de novo (branched-chain amino acids, polyamines, vitamins) [15]. For instance, SCFAs are produced from undigested carbohydrates during fermentation, of which propionate is produced mainly by phylum *Bacteroidetes*, *Lachnospiraceae*, *Negativicutes*, and *Roseburia inulinivorans* and *Ruminococcus obeaum*, while butyrate is produced by numerous species in the phylum *Firmicutes*. SCFAs regulate Treg cells’ differentiation and cytokines’ production, the activation of B cells, and antibody production; inhibit the maturation of dendritic cells; and enhance the antimicrobial function of macrophages. Tryptophan catabolites (such as indole, indolepropionic acid, and indoleacetic acid) play a role in the induction of Treg cells, CD4+ CD8+ intraepithelial lymphocytes, and inhibition of Th17 development. Similarly, secondary bile acids play a role in the induction of Treg differentiation and suppression of Th17 differentiation [15].

Several diverse factors influence the intestinal microbiota composition, including host genetics, mode of delivery, infant feeding practices and dietary patterns, use of antibiotics, geographic factors, and other environmental epigenetic exposures [9,10,14].

**Figure 1:**
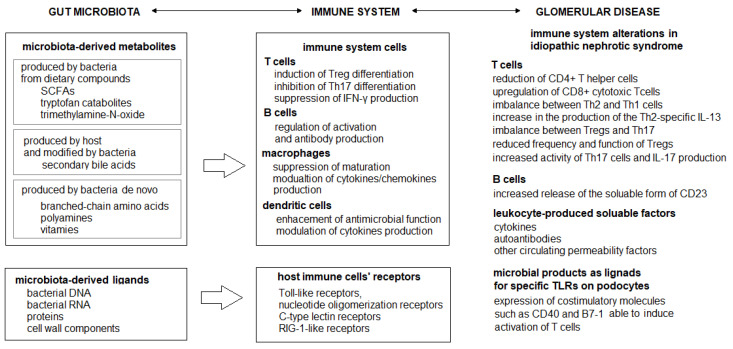
Gut microbiota impact on host immunity and immunological changes observed in idiopathic nephrotic syndrome [15,17,18].

Changes in the microbiota composition and gut microbiota-derived metabolites have been linked to the pathogenesis of several immune-related inflammatory diseases [15]. Although the interplay between the glomerulus and the immune system is still under investigation, evidence indicates that the immune system plays a significant role in the pathogenesis of non-congenital primary glomerulonephritis [18]. As microbiota-derived metabolites regulate the development and function of T cells, B cells, macrophages, and dendritic cells, these are examples of mechanisms by which gut microbiota may be involved in the pathogenesis of primary glomerulonephritis. Figure 1 illustrates the possible link between gut microbiota impact on host immunity, and immunological changes observed in idiopathic nephrotic syndrome.

## 3. Search Strategy and Data Sources

**Figure 2:**
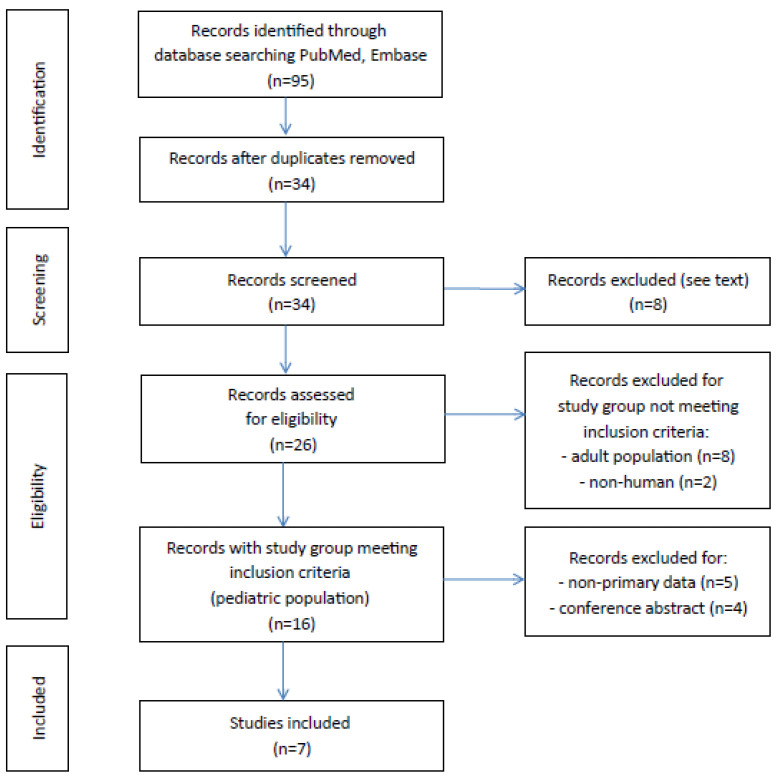
Flowchart illustrating search strategy and study selection.

A literature search of MEDLINE (PubMed) and Embase (Ovid) was conducted to identify all published articles investigating the link between gut microbiota and primary glomerulonephritis in children. The following inclusion criteria were used: a study assessing gut microbiota composition in pediatric patients with diagnosed primary glomerulonephritis, written in English, and with full-text available. Search strategies used the following expressions: “(microbiota) AND (idiopathic nephrotic syndrome) AND (children)”, “(microbiota) AND (membranous nephropathy) AND (children)”, “(microbiota) AND (immunoglobulin a nephropathy) AND (children)”, “(microbiome) AND (idiopathic nephrotic syndrome) AND (children)”, “(microbiome) AND (membranous nephropathy) AND (children)”, “(microbiome) AND (immunoglobulin a nephropathy) AND (children)”, “(dysbiosis) AND (idiopathic nephrotic syndrome) AND (children)”, “(dysbiosis) AND (membranous nephropathy) AND (children)”, and “(dysbiosis) AND (immunoglobulin a nephropathy) AND (children)”. A total of 95 articles were identified in the databases browsed, 61 studies were excluded because they were duplicated, 7 were excluded following abstract and/or title review, and 1 was excluded because it was written in Chinese (Figure 2). After this step, 10 records were excluded because the study population did not meet the inclusion criteria. Of 16 records of studies on the pediatric population, 11 contained original primary data, but 4 were excluded for being a conference abstract. As a result, 7 articles with full-text available met the inclusion criteria. We found no studies investigating gut microbiota composition in pediatric patients diagnosed with membranous nephropathy or IgA nephropathy.

## 4. Idiopathic Nephrotic Syndrome

Idiopathic nephrotic syndrome (INS) is a common childhood disease with an estimated incidence of 1.15 to 16.9 per 100,000 children, depending on the geographical region [19]. In children, the most common histological type of INS is minimal change disease accounting for 70–90% of cases [20]. Its pathogenesis remains elusive; however, it is attributed mainly to dysfunction or dysregulation of T cells, the presence of a circulating glomerular permeability factor, and according to recent studies, possibly also the dysfunction of B cells [18,21]. Growing evidence indicates a link between the gut microbiome and immune-mediated diseases, including the impact of dysbiosis on INS onset or recurrence. The suggested mechanism is connected with the observation that changes in the gut microbiome composition resulting in decreased SCFAs production may cause Treg abnormalities [22]. Butyric acid, by strong inhibition of histone deacetylase, enhances histone acetylation in the promoter and enhancer regions of the Foxp3 [23], a transcription factor specially expressed in Treg cells, that controls the expression of crucial immune-regulatory genes [24].

This hypothesis is supported by Tsuji et al. who observed a reduced proportion of butyric acid bacteria and lower fecal butyric acid quantities in pediatric patients with relapsing INS concomitantly with decreased circulatory Treg cells. They reported a lower amount of butyric acid-producing bacteria, such as *Clostridium* clusters IV, XIVa, *Eubacterium* spp, and *Butyrivibruio* spp., in fecal samples in children with relapsing INS compared to healthy controls, but not in the non-relapsing group. The comparison of fecal butyric acid quantities in feces confirmed that it was significantly lower only in patients with relapsing INS. There were no differences in other fecal organic acid quantities between groups [25].

A possible association between Treg cells, gut microbiota, and INS during the disease’s onset and frequent relapses in the follow-up was investigated in another study by Tsuji et al. [26]. A significant reduction in the proportion of butyrate-producing bacteria genera in fecal samples of children with frequent relapses compared to healthy controls was shown, but not between non-relapsing patients and healthy controls. In addition, the rate of increase in Tregs response to glucocorticosteroid therapy was higher in the non-relapsing group than in the frequently relapsing group [26].

According to Yamaguchi et al., there was no difference in the diversity of the gut microbiota in the Shannon index or the number of species between healthy controls and children with INS before treatment [27]. However, the percentage of butyrate-producing bacteria species in children with INS at the onset of the diseases was significantly lower than in healthy controls (2.2% vs. 6.7%) [27]. The Shannon index is a quantitative indicator of the number of different bacteria present in a fecal sample, taking into account the uniformity in the distribution of these bacteria in these species [28].

Kang et al. assessed the alterations of gut microbiota in a group of 20 children with nephrotic syndrome before and after 4-week initial therapy with glucocorticosteroids and did not show changes in richness or diversity of intestinal microbiota [29]. However, the analysis of fecal samples after initial therapy showed an increase in the relative abundance of *Deinococcus-Thermus* and *Acidobacteria* at the phylum level. The compositional changes at the genus level also were observed, with an increase in the amount of *Romboustia*, *Stomatobaculum*, *Cloacibacillus*, *Howardella*, *Mobilitalea*, *Deinococcus*, *Paracoccus*, *Stenotrophomonas*, *Gp1*, *Kocuria*, *Pseudomonas*, *Acinetobacter*, *Brevundimonas*, and *Lactobacillusbacteria*, and decrease in the abundance of *Finegoldia* and *Corynebacterium*. The significant increase in the abundance of SCFA-producing bacteria, such as *Romboustia*, *Stomatobaculum*, and *Cloacibacillus* after initial therapy, supports the hypothesis of gut microbiota impact on Treg induction and differentiation and its link with the onset or exacerbation of glomerulonephritis mediated by SCFAs. Moreover, specific structural changes of microbiota result in functional profile change, in particular by weakening the selenocompound metabolism, isoflavonoid biosynthesis, and phosphatidylinositol signaling system, the pathways that are important in antioxidant defense. The diminished selenocompuund metabolism might help maintain the appropriate level of selenoproteins, which are the key enzymes in redox homeostasis and therefore might contribute to the remission of proteinuria after initial therapy. In contrast, decreased isoflavonoid biosynthesis and phosphatidylinositol signaling may be associated with higher relapse occurrence in children with INS [29].

Likewise, Tingting et al. suggest that gut microbiota changes may be correlated with Th17/Treg cell imbalance and play a role in the pathogenesis of INS in children [30]. In children with a new onset of INS, the counts of *Lactobacillus*, *Bifidobacteria*, and *Escherichia coli (E.coli)* were lower than in healthy controls and significantly increased after treatment but did not reach the level of healthy controls. After treatment, a decrease in the proportion of Th17 cells and an increase in the proportion of Treg cells were observed. Further, the ratio of Th17/Treg cells, which was increased before treatment in children with INS, recovered to the normal level after treatment. The authors reported a negative correlation between Th17/Treg cell ratio and the count of *E.coli* in the study group. The counts of *Lactobacillus* and *Bifidobacteria* or the *Bifidobactera/E.coli* ratio were not correlated with the Th17/Treg cell ratio [30].

Notably, according to Szlachciński et al., pediatric patients with INS in remission during immunosuppressive therapy present unfavorable changes in gut microbiota [31]. The results of a cross-sectional study in a group of 44 children with INS indicate that immunosuppressive treatment might result in changes in microbiota composition. The alterations in gut microbiota homeostasis in patients with INS may be related not only to the disease itself, but also might be secondary to the immunosuppressive treatment. Moreover, different therapeutic options present dissimilar influences on intestinal microbiota profile. For instance, the most profound changes in gut microbiota have been observed for children treated with cyclosporine A who had the lowest total number of bacterial colonies in fecal samples and significantly higher amount of *Candida* sp. colonies [31].

It should be underlined that children with INS are frequently treated with different antibiotics because of increased susceptibility to infections during immunosuppressive therapy, and antibiotics are known for their influence on intestinal microbiota [32].

The current evidence on gut microbiota dysbiosis in children with INS compared to healthy controls is summarized in Table 1. The changes in microbiota composition during the immunosuppressive therapy are briefly reported in Table 2.

## 5. Membranous Nephropathy

Membranous nephropathy is the most common cause of idiopathic nephrotic syndrome in adults [33]. In children, membranous nephropathy more frequently is secondary to systemic diseases, such as systemic lupus erythematosus or hepatitis B [34]. The etiology of primary membranous nephropathy is associated with the production of autoantibodies directed against podocyte antigens, primarily M-type phospholipase A2 receptor (PLA2R) or thrombospondin type-I domain-containing 7A (THSD7A) [34]. In some cases, when the disease is not presumed to be linked with systemic diseases or secondary causes, the target antigen may remain unknown.

It hypothesized that gut dysbiosis may be related to idiopathic membranous nephropathy due to autoantibody production induced by clonal expansion of B cells promoted by mucosa-associated lymphoid tissue [4], decreased SCFAs production [35], or increased generation of pro-inflammatory cytokines due to activation of the NF-κB pathway by lipopolysaccharide produced by *Escherichia-Shigella* and *Bacteroides* [4].

Although the changes in gut microbiota structure were identified in adult patients with idiopathic nephrotic syndrome and histopathological diagnosis of membranous nephropathy, there is a lack of similar studies conducted among children with primary membranous nephropathy.

## 6. Immunoglobulin A Nephropathy

IgA nephropathy (IgAN) is the most common primary glomerulopathy in general population [36]. It is characterized by the deposition of immunological complexes in glomeruli, which are composed of galactose-deficient IgA1 (Gd-IgA1), IgG autoantibodies against the hinge region of Gd-IgA1, and complement component C3 [36,37]. Because IgA is primarily produced by gastrointestinal lymphoid tissue in response to microorganisms, the role of the gut axis in the pathology of IgAN is evident [16]. Moreover, the structural aspects of glomerular IgA deposits and circulating IgA immune complexes in IgAN indicate an intestinal origin [7].

We found no studies regarding dysbiosis and pediatric IgAN patients. However, a possible causal link between dysbiosis and IgAN is suggested due to increased intestinal permeability [16], decreased production of SCFAs [38], and mucosal hyperresponsiveness, resulting in an abnormal production of Gd-IgA1 [39]. The mucosal hyperresponsiveness may result from a significant difference in the amount of intestinal-activated B lymphocytes observed in IgAN patients [39].

## 7. Therapeutical Gut Microbiota Modifications

The possible usage of gut microbiome modifications might be considered supportive therapy in immune-mediated diseases due to the crosstalk between intestinal bacteria and host immunity mediated by different complex interactions between intestinal bacteria metabolites [40]. Recent studies reported the occurrence of dysbiosis characterized by decreased abundance of SCFA-producing bacteria in adults and children with primary glomerulonephritis [22,25,35,38]. Therefore, the intervention to reshape or change the gut microbiota composition aiming to influence the immune responses might be an additional component in supportive therapy. These interventions include the use of microbial supplements, such as probiotics or synbiotics, or foods or substrates (diet or prebiotics) [41]. The number of studies focusing on therapeutic modification of the gut microbiota in pediatric patients with primary glomerulopathies is relatively limited (Table 3) [27,42].

### 7.1. Probiotics

Potentially beneficial might be the use of *Lactobacillus plantarum* in children and adolescents with INS. A randomized, double-blind, placebo-controlled clinical trial conducted by Fortes et al. suggests that the administration of this probiotic strain might have an immunomodulatory and hypolipidemic effect in pediatric patients with INS and dyslipidemia [43]. Among probiotic-treated children, the tendency to reduce TNF-α levels and increase IL-10 levels was observed. However, the inclusion criteria were met only by four patients; thus, the results should be interpreted with caution.

The possible use of a probiotic containing SCFA-producing bacteria in the reduction of relapses of INS in children was evaluated by Yamaguchi et al. [27]. The study group consisted of 20 children with INS (median age 5.3 years) during remission. The intervention was a daily administration of preparation of *Clostriudium butyricum.* Two-year follow-up revealed a significantly lower annual frequency of INS relapse in the probiotic treatment group. Additionally, the number of patients who started rituximab therapy because of frequent relapses was significantly higher in the non-probiotic treatment group. In addition, in probiotic-treated patients, a significant increase in the relative abundance of butyrate-producing bacteria and blood Treg cell counts were observed.

Further studies are needed to evaluate the possible use of probiotics in the therapy of INS or other primary glomerulonephrites. The questions to answer are the choice of a specific probiotic strain, the optimal dosage, and the duration of the therapy.

It should be underlined that immunosuppressive therapy in pediatric patients with INS might also impact the gut microbiota composition. Kang et al. indicate that glucocorticosteroid therapy could disrupt gut microbiota, but the impact of other substances used together with glucocorticosteroids, such as vitamin D3 and calcium carbonate, cannot be excluded, and together these agents may have a synergistic effect [29]. Furthermore, other immunosuppressive drugs, including cyclosporine A, may cause disturbances in intestinal microbiota composition [31].

### 7.2. Dietary Interventions

Diet is one of the key elements that modulate gut microbiota composition and function [44,45]. Some research suggests that specific diets may alleviate the imbalance in intestinal microbiota [46]. In search of therapeutic options for patients with nephrotic syndrome, especially those with steroid-dependent or steroid-resistant nephrotic syndrome, the possible impact of dietary modifications on a disease course has been studied [46]. Among elimination diets, gluten and dairy restrictions have been linked with a significant reduction of proteinuria in patients with steroid-sensitive, steroid-dependent, or steroid-resistant nephrotic syndrome [46,47]. Although most of these observations were based upon clinical cases of patients with food sensitivities [46], the proposed mechanisms are alterations in the intestinal microbiota resulting in changes in the production of inflammatory mediators that may act as glomerular permeability factors, or direct effect of gluten-free diet on podocyte structure [48].

According to a prospective study that assessed the immunological impact of a gluten-free and dairy-free diet in a group of children with steroid-resistant nephrotic syndrome, dietary interventions had an anti-inflammatory effect characterized by an increase in the Treg and T helper cells ratio and a decrease in pro-inflammatory cytokines, such as TNF-α and IL-8 [42]. In addition, after four weeks of a gluten-free and dairy-free diet, there was a significant change in gut microbiota composition with an increase in abundance of *Bacteroidetes*, *Lachnospira*, and *Faecalibacterium*. Observed changes in the intestinal microbiota structure presumably present a favorable immune-regulatory phenotype [42].

Although the results might be promising and worth consideration, the application of a restricted diet in children should be reserved for specific cases with careful risk–benefit assessment.

### 7.3. Fecal Microbiota Transplantation

Another treatment option aiming to reshape the composition of gut microbiota is fecal microbiota transplantation (FMT) [49] Its effectiveness has been observed in the treatment of recurrent and refractory *Clostridium difficile* infection, both in pediatric and adult patients [49,50,51]. It is presumed a promising therapy not only in gastrointestinal tract diseases, such as inflammatory bowel disease or irritable bowel syndrome, but also in several autoimmune diseases, metabolic syndrome, and neurological disorders [49,50,51,52]. Only a few clinical cases reported the usage of FMT in the treatment of adult patients with primary glomerulopahties [53,54]. Partial clinical remission was observed in two female patients with refractory IgAN after FMT [53]. FMT application in a male patient suffering from chronic diarrhea and membranous nephropathy alleviated gastrointestinal symptoms and improved renal function [54]. Additional studies are needed to assess the safety, effectiveness, and short- and long-term effects of FMT in non-gastrointestinal diseases, including glomerular diseases [50,51].

## 8. Conclusions and Directions for Further Research

Mounting evidence indicates a possible link between gut microbiota and kidney diseases, including immune-mediated glomerulonephritis [1,2,16,55]. Still, little is known about the correlation between the alterations of gut microbiota and the onset or relapse of glomerular diseases in the pediatric population. Few studies aimed to assess the compositional changes of gut microbiota in children with idiopathic nephrotic syndrome [25,26,27,28,31,56]. However, studies regarding gut dysbiosis and nephropathy IgA or primary membranous nephropathy in pediatric patients are lacking, indicating the direction for further research.

It is unclear if observed changes in intestinal microbiota structure in patients with glomerular disease are related to the disease itself or are secondary to the immunosuppressive treatment [29,30,31]. Other drugs frequently used in patients treated with glucocorticosteroids, including antibiotics or proton pump inhibitors, influence the gut microbiota composition and function as well, and their interference should also be considered [27,32,57,58].

Among the limitations connected with the research in pediatric patients, the small study group is one of the important aspects. Furthermore, controlling for confounding factors that might affect the gut microbiota composition is challenging. Therefore, each study should include the assessment of the child’s delivery mode, feeding practices in infancy, dietary habits, and the use of antibiotics and non-antibiotic drugs.

Since geographic factors may influence the intestinal microbiota composition, geographical bias should be considered while analyzing the possible link between gut microbiota and disease onset or relapse in different populations [11,59,60,61]. Most of the studies we referred to were conducted in Asia, in particular in Japan [25,26,27] and China [29,30]. Other studies focusing on pediatric patients with nephrotic syndrome were conducted in Poland [31] and the United States of America [44]. Notably, there are significant differences in incidence and prevalence of nephrotic syndrome in children according to geographical region [19].

Current genome-wide association studies (GWAS) and sequencing studies were applied to primary glomerular disorders, providing novel insights into the genetic architecture of IgA nephropathy, membranous nephropathy, and steroid-sensitive nephrotic [62]. According to Sanchez-Rodriguez et al., the genetic risk correlates strongly with variation in regional pathogens, suggesting a potential role of host–intestinal pathogen interactions in shaping the disease susceptibility, especially in IgA nephropathy [62]. Moreover, several GWAS studies indicate that host genetics affects the structure and composition of intestinal microbiota [63,64]. Thus, further research is needed to evaluate the link between host genetics and epigenetics, including the impact of intestinal microbiota and susceptibility to primary glomerular diseases.

Recent advances in molecular science enabled precise qualitative and quantitative assessment of gut microbiota composition, mainly due to the 16S ribosomal RNA sequencing technique [65]. From a theoretical point of view, despite the compositional analysis of the gut microbiome, characterization of related functional shifts and changes in the gut microbiota-derived metabolites are basic in understanding the interplay between gut dysbiosis, immune system, and glomerulopathies [12].

Finally, there are clinical implications of the observed correlations. Further directions include seeking a new therapeutic option as supportive therapy and the possible use of gut microbiota manipulation in the regulation of the host immune response. Forthcoming randomized clinical trials could address the role of probiotics and dietary interventions via gut microbiome modulation in the reduction of proteinuria or maintaining remission in pediatric patients with idiopathic nephrotic syndrome or IgA nephropathy.

## Figures and Tables

**Table 1:** Gut microbiota alterations in children with idiopathic nephrotic syndrome (INS) compared to healthy controls (HC).

Study	Country	Study Group	Healthy Controls	Microbiota Assessment Methods and Samples Collection Time	Results
Tsuji et al. [25]	Japan	children with INS N =12 (male: female = 7:5) divided into relapsing group (R) = 8; median age 3.0 years non-relapsing group (NR) = 4; median age 4.3 years	N = 11 (male: female = 5:6) median age 5.1 years	Fecal samples collection and 16S rRNA sequencing Fecal butyric acid measured using high-performance liquid chromatography Fecal samples from INS patients obtained before starting therapy	Lower proportion of butyric acid-producing bacteria and fecal butyric acid quantities in R group than in HC (*p* = 0.0013 and *p* = 0.042 respectively).
Tsuji et al. [26]	Japan	children with INS N = 25 (male: female = 20:5) median age 4.0 years divided into non-relapsing group (NR) = 8, median age 5.4 years, frequently relapsing group (FR) = 17, median age 3.5 years	N = 20 (male: female = ns) median age 4.0 years	Fecal samples collection and 16S rRNA sequencing Fecal samples from INS patients obtained before starting therapy	Different distribution of bacteria in FR group compared to HC and NR group. Reduced proportion of butyric acid-producing bacteria in the FR group compared to HC (*p* = 0.0031).
Yamaguchi et al. [27]	Japan	children with INS N = 20 (male: female = 15:5) median age 5.3 years	N = 21 (male: female = ns) median age 4.0 years	Fecal samples collection and 16S rRNA sequencing Fecal samples from INS patients obtained at the onset of INS	The percentage of butyrate-producing bacteria was significantly lower in study group at INS onset compared with HC (*p* = 0.024).
Tingting et al. [30]	China	children with INS N = 29 (male: female =18:11) mean age 7.23 ± 2.06 years	N = 15 (male: female = 10:5) mean age 7.31 ± 2.12 years	Fecal samples collection and 16S rRNA sequencing Fecal samples form INS patients obtained before treatment or remission after treatment	Decreased counts of *Lactobacillus*, *Bifidobacteria*, and *E.coli* before treatment in study group compared to HC (*p* < 0.05). Decreased *Bifidobacteria* and *E.coli* ratio at INS onset.
Szlachciński et al. [31]	Poland	children with INS N = 44 (male: female = 26:18) median age ns divided according to treatment protocols: - group A (CsA ± GCS) N = 18 (male: female = 11:7) age 2–14 years - group B (GCS) N = 17 (male: female = 9:8) age 2–17 years - group C (CYC + GCS) N= 9 (male: female = 6:3) age 3–12 years	N = 20 (male: female = 13:7) age 2–15 years	Fecal samples collection and culture (KyberStatus and KyberMyk test) Fecal samples form INS patients obtained once during the therapy	Lower total number of bacterial colonies in group A (*p* < 0.001) and group B (*p* = 0.04) compared to HC. Lower number of *Bifidobacterium* colonies in group C compared to HC (*p* = 0.01). Higher amount of *Candida* sp. colonies in group A compared to HC (*p* = 0.01).

INS—idiopathic nephrotic syndrome, ns—not specified, R—relapsing group, NR—non-relapsing group, FR—frequently relapsing group, HC—healthy control, N—number of participants, GCS—glucocorticosteroids, CsA—cyclosporine A, CYC—cyclophosphamide, rRNA—ribosomal RNA.

**Table 2:** Gut microbiota alterations in children with idiopathic nephrotic syndrome (INS) during immunosuppressive treatment.

Study	Country	Study Group	Microbiota Assessment Methods and Samples Collection Time	Results
Kang et al. [28]	China	children with INS N = 20 (male: female = 15:5) mean age 3.5 ± 2.1 years	Fecal samples collection and 16S rRNA sequencing Fecal samples collected before and after 4-week initial therapy	The richness and diversity of gut microbiota were similar before treatment and after 4-week initial therapy and achieved complete remission. The abundance SCFA-producing bacteria including *Romboutsia*, *Stomatobaculum*, and *Cloacibacillus* increased after initial therapy (*p* < 0.05).
Tingting et al. [30]	China	children with INS N = 29 (male: female = 18:11) mean age 7.23 ± 2.06 years	Fecal samples collection and 16S rRNA sequencing Fecal samples form INS patients obtained before treatment or remission after treatment	Counts of *Lactobacillus*, *Bifidobacteria*, and *E.coli* recovered after treatment but did not reach the normal level. *Bifidobacteria* and *E.coli* ratio increased after treatment (*p* < 0.05).
Szlachciński et al. [31]	Poland	children with INS N = 44 (male: female = 26:18) median age ns divided into groups according to treatment protocols: - group A (CsA ± GCS) N = 18 (male: female = 11:7) age 2–14 years - group B (GCS) N = 17 (male: female = 9:8) age 2–17 years - group C (CYC + GCS) N = 9 (male: female = 6:3) age 3–12 years	Fecal samples collection and culture (KyberStatus and KyberMyk test) Fecal samples form INS patients obtained once during the therapy	Lower total number of bacterial colonies in group A compared to group B (*p* = 0.007) and C (*p* = 0.04).

INS—idiopathic nephrotic syndrome, ns—not specified, N—number of participants, GCS—glucocorticosteroids, CsA—cyclosporine A, CYC—cyclophosphamide, rRNA—ribosomal RNA, SCFA—short-chain fatty acids.

**Table 3:** The therapeutical gut microbiota modifications in children with idiopathic nephrotic syndrome (INS).

Study	Country	Study Group	Microbiota Assessment Methods and Sample Collection Time	Intervention	Results
Yamaguchi et al. [27]	Japan	children with INS N = 20 (male: female = 15:5) median age 5.3 years Probiotic-treated group: N = 10, median age 6.4 years Non-probiotic-treated group: N = 10, median age 4.7 years	Fecal sample collection and 16S rRNA sequencing Fecal samples from INS patients obtained at the onset of INS and during treatment with probiotics	Oral administration of butyrate-producing bacteria (C.butyricum MIYAIRI) started at the end of the 8-week steroid administration dosing 3 g/day median period of intervention 25 months (range from 7 to 46 months)	The percentage of butyrate-producing bacteria increased after probiotic treatment (*p* = 0.017). Probiotic-treated patients experienced fewer INS relapses per year compared with non-probiotic-treated patients (*p* = 0.016).
Perez-Saez et al. [42]	USA	children with steroid-resistant nephrotic syndrome (SRNS) N = 16 (male: female =8:8) mean age 7.0 ± 5.3 years	Fecal sample collection and 16S rRNA sequencing Fecal samples collected at baseline and after the intervention (day 54)	A 4-week summer camp implementing a strict gluten-free and dairy-free diet (GF/DF diet)	Increased fraction of *Bacteroides*, *Lachnospira*, and *Faecalibacterium* after the intervention. GF/DF diet promoted a favorable microbiome modification with potential immune-regulatory phenotype. Overall, 2 out of 16 participants achieved complete remission in proteinuria after 4 weeks on GF/DF diet. Both participants experienced recurrence in proteinuria after returning to unrestricted diet, after which they immediately went back to a GF/DF diet, achieving again a sustained remission in proteinuria.

INS—idiopathic nephrotic syndrome, N—number of participants, SRNS—steroid-resistant nephrotic syndrome, rRNA—ribosomal RNA, GF/DF diet—gluten-free and dairy-free diet.

## Data Availability

Not applicable.
